# Increased Kappa/Lambda Hybrid Antibody in Serum Is a Novel Biomarker Related to Disease Activity and Inflammation in Rheumatoid Arthritis

**DOI:** 10.1155/2016/2953072

**Published:** 2016-04-06

**Authors:** Lang Yi, Mingju Hao, Tian Lu, Guigao Lin, Lida Chen, Ming Gao, Gaowei Fan, Dong Zhang, Guojing Wang, Xin Yang, Yulong Li, Kuo Zhang, Rui Zhang, Yanxi Han, Lunan Wang, Jinming Li

**Affiliations:** ^1^National Center for Clinical Laboratories, Beijing Hospital, Beijing 100730, China; ^2^Graduate School, Peking Union Medical College, Chinese Academy of Medical Sciences, Beijing 100730, China; ^3^China-Japan Friendship Hospital, Beijing 100730, China; ^4^Department of Rheumatology, Beijing Hospital, Beijing 100730, China

## Abstract

The *κ*/*λ* hybrid antibodies in normal human serum were reported recently, but their clinical relevance has not yet been explored. Rheumatoid arthritis (RA) is one of the major joint diseases, and the early diagnosis and treatment of RA remain a challenge. Here, we developed a double-sandwich enzyme-linked immunosorbent assay system to quantify relative serum *κ*/*λ* hybrid antibody levels in RA patients, osteoarthritis (OA) patients, and healthy controls (HC) in order to assess their potential use as a serological biomarker of early disease and clinical activity and to preliminarily investigate their immunomodulatory roles in RA. Surprisingly, we found that *κ*/*λ* hybrid antibody was markedly increased in both early and established RA. Serum *κ*/*λ* hybrid antibody levels were significantly correlated with clinical indexes and inflammatory markers in RA. Further analysis showed a positive correlation between *κ*/*λ* hybrid antibody levels and the 28-joint disease activity score (DAS28). In conclusion, serum *κ*/*λ* hybrid antibodies in RA were identified for the first time. High levels of *κ*/*λ* hybrid antibody may be a useful tool in distinguishing early RA from OA and HC. We suggest *κ*/*λ* hybrid antibody as a marker for disease activity. The increased *κ*/*λ* hybrid antibodies were associated with inflammatory conditions in RA.

## 1. Introduction

Rheumatoid arthritis (RA) is an autoimmune inflammatory disease hallmarked by a chronic form of arthritis, which often eventually leads to tissue degradation, irreversible joint damage, and severe disability. RA is an increasingly important issue in current aging populations and affects up to 1.1% and 0.37% of the populations of Western countries and China, respectively [1, 2]. The exact etiopathogenesis of RA is still uncertain. Previous studies demonstrated that early diagnosis and treatment for patients with RA are beneficial to reducing of progressive joint injury, whereas a few months' delay of treatment from the onset of symptoms would decrease the potential ability of the traditional single-drug strategy to induce remission in early RA (ERA) [3, 4]. Under this circumstance, biomarkers for early diagnosis are of great significance. However, the early diagnosis of RA remains a challenge. Currently, several biomarkers such as genetic accessibility, miRNA, and gene expression have been described but are either lacking in specificity or impractical for routine clinical practice [5–7]. A serological test which can preferably detect and distinguish various types of arthritis early is required.

It is generally accepted that mature plasma cells produce symmetrical antibodies with a single type of L chain, either kappa (*κ*) or lambda (*λ*) [8]. However, a small fraction of *κ* ^+^ *λ* ^+^ dual receptor B cells are described in the peripheral repertoire of normal human subjects and myelomas patients, which express both *κ* and *λ* on the same cell surface and produce immunoglobulin that contains both the *κ* and *λ* chains in the same molecule; these are termed *κ*/*λ* hybrid antibody [9–11]. These *κ* ^+^ *λ* ^+^ dual receptor B cells are further demonstrated in a mouse model and may be related to autoantibody production [12, 13]. Recently, studies reported that IgG4 can form bispecific antibody through dynamic Fab-arm exchange as described in our previously published work about Hashimoto thyroiditis and others about allergic diseases [14, 15]. Subsequently, polyclonal IgG4 *κ*/*λ* hybrids, which are produced based on the postsecretion modification process of half-molecular exchange, were observed in normal human serum [16]. This is the first study to focus on *κ*/*λ* hybrid antibody detection in normal human serum. However, the clinical relevance of such molecules has not been clarified or discussed. Autoimmune disease is characterized by immune dysfunction resulting in excessive autoantibody production. Taken together, we hypothesised that changes in *κ*/*λ* hybrid antibody levels might be linked to the inflammatory state of chronic autoimmune disease.

Therefore, the purpose of this study was to discuss the hypothesis that altered *κ*/*λ* hybrid antibody levels in RA patients may act as a new biomarker of RA and to preliminarily discuss its potential immunomodulatory role in autoimmune disease. Furthermore, we investigated whether *κ*/*λ* hybrid antibody levels would be a marker of disease activity in RA.

## 2. Materials and Methods

### 2.1. Collection of Sera and Characteristics of Patients with RA

Serum samples and clinical details of 58 RA patients were obtained from the Department of Rheumatology, Beijing Hospital. All of the patients fulfilled the American College of Rheumatology (ACR)/European League against Rheumatism (EULAR) 2010 classification criteria for RA [17]. Sixteen ERA patients were newly diagnosed and previously untreated with a symptom duration < 6 months. Serum samples of established treated RA were obtained from 42 patients with a disease duration of 8.18 ± 8.12 years. For comparison, serum samples from 20 patients with osteoarthritis (OA) and 20 healthy controls (HC) were also collected from Beijing Hospital and tested. The clinical characteristics of the patients and HC are outlined in Table 1. The serum samples we used for hybrid antibody assessment were collected at the same time point as the clinical laboratory tests were carried out. Serum samples were stored at −80°C until analysis. Written informed consent was obtained from all participants and the study was approved by the ethics committee of the National Center for Clinical Laboratory and was conducted in accordance with the guidelines of the Declaration of Helsinki.

### 2.2. Measurement of Serum *κ*/*λ* Hybrid Antibody Levels

Serum *κ*/*λ* hybrid antibody levels were measured by using a double-sandwich enzyme-linked immunosorbent assay (ELISA) system. In brief, 96-well assay plates (Nunc MaxiSorp, Denmark) were coated with mouse anti-human lambda chain monoclonal antibody (Abcam, China; 100 *μ*L/well at 5 *μ*g/mL) diluted in 0.05 M sodium carbonate buffer (pH 9.6) overnight at 4°C. Then, the plates were washed with washing buffer (0.05% v/v Tween 20 in PBS (PBST)) and blocked by the same buffer supplemented with 1% (m/v) gelatin (Sigma-Aldrich, USA; 300 *μ*L/well) for 2 hours at 37°C. After washing, the serum samples were diluted 10,000-fold in serum diluent (1% m/v gelatin in PBST) and added to the wells (100 *μ*L/well) and then incubated for 1 hour at 37°C, followed by five washes. Thereafter, 100 *μ*L of the mouse anti-human kappa light-chain HRP conjugated antibodies (Abcam, China) diluted 1 : 2000 in PBST-1% gelatin was added to each test well. After incubation (1 hour at 37°C), the plates were thoroughly washed and developed with 100 *μ*L/well tetramethylbenzidine (Sigma-Aldrich, USA) for 10 min at 37°C in the dark. The reaction was stopped with sulfuric acid (0.5 M, 50 *μ*L/well), and absorbance was read at 450 nm with wavelength correction at 620 nm by using an ELISA reader (Thermo Fisher Scientific, USA). All serum samples were tested in duplicate, and the results were averaged. The working concentration of each reagent of the test was determined based on chessboard titrations to optimize the assay performance [18].

In order to compare different results, a reference standard was included in all the plates to construct a standard curve. As no defined standards are available, a serum mixture of 20 healthy sera was used as the standard reference serum, which was arbitrarily assigned to contain four units of *κ*/*λ* hybrid antibodies per milliliter (AU/mL). The standard reference serum was split and stored at −80°C until used. A twofold serial dilution (1 : 1000 to 1 : 128000) of the standard reference serum in the dilution buffer was measured in parallel with all samples in all the ELISA studies. Standard curves were developed by using the four-parameter logistic-log curve fitting method. *κ*/*λ* hybrid antibody units of the serum samples were then calculated from their optical density (OD) values by using the parameters estimated from the standard curve. Serum samples need to be tested again with a higher dilution when its absorbance value exceeds the linear portion of the standard curve. Variation between the plates never exceeded 5%.

To exclude false-positive results caused by RF, four RF positive RA serum samples were treated with dithiothreitol (DTT) (Amresco, USA) [19] or concanavalin (Sigma-Aldrich, USA) [20] to remove IgM RF. The control serum samples were treated with PBS or solvent. The effect of IgM RF elimination was measured by using a commercial ELISA kit (AESKU, Germany) according to the manufacturer's instructions. The levels of *κ*/*λ* hybrid antibodies were then tested and compared with the untreated group.

### 2.3. Measurement of Inflammation Markers

To investigate the relationship between the *κ*/*λ* hybrid antibodies and inflammation status of RA, a panel of well-studied inflammatory factors involved in RA (i.e., interferon-*γ* (IFN-*γ*), IL-6, IL-10, and TNF-*α*) were measured. Quantification of the cytokines was accomplished by using quantitative commercial ELISA kits (R&D systems, USA). In brief, standards with known content, controls (negative control, positive control, and cutoff calibrator), and serum samples were tested at the same time according to the manufacturer's instructions. The concentration of each marker was calculated from its corresponding standards.

### 2.4. Laboratory Investigation and Assessment of Disease Activity

To analyze the relationship between *κ*/*λ* hybrid antibodies and other RA-related laboratory biomarkers in order to confirm their clinical significance, data on ACPA (anti-citrullinated protein antibodies), RF, ESR, CRP, D-dimer, and routine biochemistry of each patient were collected from medical records. ACPA level was measured by using the second generation CCP2 antibody ELISA kit (Immunoscan CCPlus; Euro-Diagnostica). ESR (mm/h) was measured by using the Westergren method. CRP and RF were assayed by using immunonephelometry (Nephelometer Analyzer II, Siemens), by using reagents and controls supplied by the manufacturer.

The 28-joint disease activity score (DAS28) was used as a measure of RA disease activity and was measured according to the disease activity score in 28 joints by using the number of swollen and tender joints, the ESR, and the patient's global visual analogue scale (VAS) score [21]. Categories of disease activity based on the DAS28-ESR were defined as follows [22]: remission, DAS28-ESR < 2.6; low, DAS28-ESR between 2.6 and 3.2; moderate, DAS28-ESR between 3.2 and 5.1; and high, DAS28-ESR > 5.1.

### 2.5. Detection of Serum Total IgG4

To determine whether *κ*/*λ* hybrid antibody levels and serum total IgG4 levels were correlated, serum total IgG4 level was measured by using immunonephelometry (Nephelometer Analyzer II, Siemens) with commercial kits (N Latex IgG4, Siemens). The reference interval for adults was 80–1,350 mg/L according to the manufacturer's instructions.

### 2.6. Statistical Analysis

The ELISA results of *κ*/*λ* hybrid antibodies detection were analyzed by a four-parameter logistic-log curve fitting program (ELISA v. 2.15, USA) and switched to arbitrary units (units per milliliter, AU/mL). Statistical analysis was performed by using SPSS 19.0 (SPSS Inc., USA) and GraphPad Prism Version 6.0 (GraphPad, USA). Data were expressed as mean ± SD values, or mean ± SEM, as appropriate. Nonparametric analyses with the Mann-Whitney *U* test were performed to compare data between groups. Correlations between any two variables were determined by using the Spearman rank correlation coefficients. A two-sided *p* < 0.05 was considered significant. Statistically significant differences are indicated with *∗*, *∗∗*, and *∗∗∗* for *p* < 0.05, 0.01, and 0.001, respectively. The receiver operating characteristic (ROC) curve was generated by plotting the sensitivity against one-specificity, and the area under the curve (AUC) with 95% CI was calculated.

## 3. Results

### 3.1. Serum *κ*/*λ* Hybrid Antibody Levels Are Elevated in ERA and Established RA Compared with Those in OA and HC

**Figure 1:**
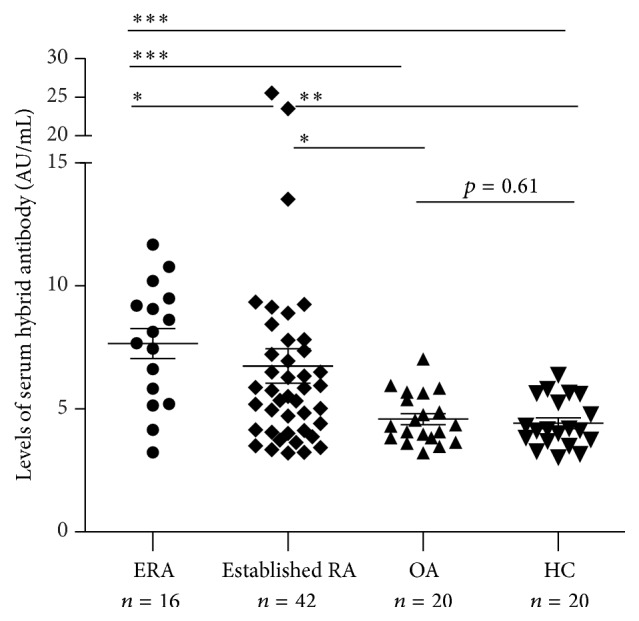
Comparison of levels of *κ*/*λ* hybrid antibody in the sera of patients with treatment naïve ERA, established RA, OA, and HC. Serum levels of *κ*/*λ* hybrid antibody were measured by enzyme-linked immunosorbent assay in patients with ERA (*n* = 16), established RA (*n* = 42), OA (*n* = 20), and HC (*n* = 20). Serum levels of *κ*/*λ* hybrid antibody were both significantly higher in patients with ERA and established RA compared with those in OA (*p* = 0.0003 and *p* = 0.0157, resp.) and HC (*p* = 0.0002 and *p* = 0.006, resp.). Concentrations of *κ*/*λ* hybrids antibody are presented in arbitrary units (AU/mL). Symbols represent individual subjects; solid line was labeled at mean with SEM. *p* values were determined using Mann-Whitney *U* test, ^*∗*^ *p* < 0.05, ^*∗∗*^ *p* < 0.01, and ^*∗∗∗*^ *p* < 0.001.

We developed a light-chain capture enzyme-linked immunosorbent assay (ELISA) for relative quantitation of *κ*/*λ* hybrid antibodies. The use of a standard serum allowed for comparison of data obtained with different ELISA plates. The AUs in the test samples were calculated from their ODs based on the four-parameter equation of the standard serum. As expected, *κ*/*λ* hybrid antibodies were successfully tested by using the detection system, and the distribution of the *κ*/*λ* hybrid antibodies among the ERA patients (*n* = 16), established RA patients (*n* = 42), OA patients (*n* = 20), and HC (*n* = 20) is shown in Figure 1. Surprisingly, the serum *κ*/*λ* hybrid antibody level in the ERA patients was 7.66 ± 2.45 AU/mL, which was significantly higher than that in patients with established RA (6.74 ± 4.56 AU/mL, *p* = 0.029), OA (4.58 ± 1.01 AU/mL, *p* = 0.0003), and HC (4.42 ± 1.00 AU/mL, *p* = 0.0002). In addition, *κ*/*λ* hybrid antibody levels were significantly higher in the serum of patients with established RA in comparison with patients with OA (*p* = 0.015) and HC (*p* = 0.006). No difference was observed between the OA and HC groups (*p* = 0.61).

**Figure 2:**
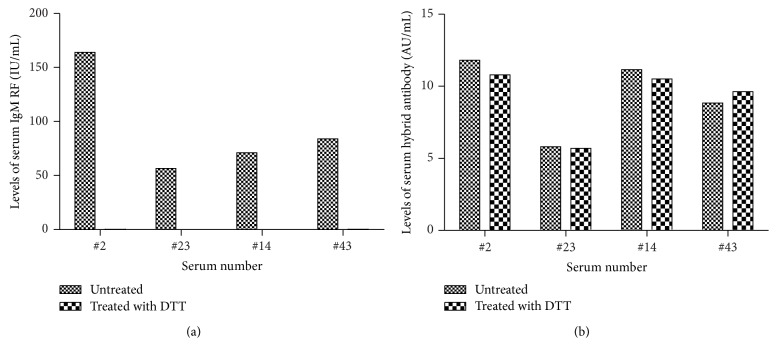
Serum IgM RF and *κ*/*λ* hybrid antibodies levels after treatment with DTT. (a) The effect of treatment with DTT on removing IgM RF. Four RF positive RA serum samples were incubated with DTT for 60 min; all or most of the IgM RF were eliminated. The concentrations of IgM RF (IU/mL) were measured by ELISA. (b) Levels of *κ*/*λ* hybrid antibodies of RA serum before and after being eliminated with IgM RF. Removing IgM RF completely showed little influence on the levels of serum *κ*/*λ* hybrid antibodies. Concentrations of *κ*/*λ* hybrid antibody are presented in arbitrary units (AU/mL).

**Figure 3:**
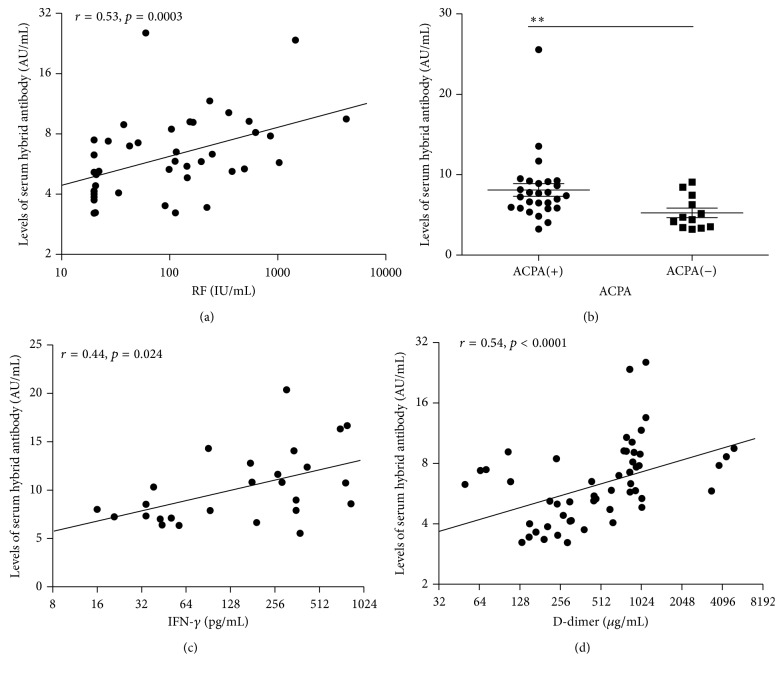
The relationship between serum levels of *κ*/*λ* hybrid antibody and RA-related clinical parameters. (a) Levels of *κ*/*λ* hybrid antibody in the serum of RA patients correlated with RF (*r* = 0.53, *p* = 0.0003). (b) Levels of *κ*/*λ* hybrid antibody were significantly higher in ACPA(+) than ACPA(−) group with *p* = 0.0065; the differences of *κ*/*λ* hybrid antibody levels between groups were assessed using the Mann-Whitney *U* test; solid line was labeled at mean with SEM, ^*∗∗*^ *p* < 0.01. (c, d) Levels of *κ*/*λ* hybrid antibody in the serum of RA patients correlated with IFN-*γ* (*r* = 0.44, *p* = 0.024) and D-dimer (*r* = 0.54, *p* < 0.0001). Concentrations of *κ*/*λ* hybrid antibody are presented in arbitrary units (AU/mL). Symbols represent individual subjects. Correlations were determined by Spearman's rank correlation coefficients with 95% confidence interval.

To exclude the false-positive results, four RF positive RA serum samples were treated with DTT or concanavalin. Results showed that after incubation with 0.01 M DTT for 60 min, all or most of the IgM RF disappeared (Figure 2(a)). However, the corresponding *κ*/*λ* hybrid antibody levels in these samples were minimally affected compared with the untreated serum (Figure 2(b)); no obvious changes were observed even when the RF concentration in the samples was high (e.g., up to 1000 IU/mL). These results were further confirmed by serum treated with concanavalin (data not shown). On the other hand, the fact that not all samples containing higher levels of RF were always *κ*/*λ* hybrid antibody high also goes against the possible interference by RF (Figure 3(a)). Thus, these combined results suggested the impossibility of false-positive result caused by RF.

### 3.2. Serum *κ*/*λ* Hybrid Antibody Levels Are Associated with RF, ACPA, IFN-*γ*, IL-6, and D-Dimer Expressions

To gain insight into the potential role of *κ*/*λ* hybrid antibodies in RA, we compared their relationship with inflammatory markers and serological parameters of RA. The serum *κ*/*λ* hybrid antibody levels in the RA patients were significantly correlated with the clinical parameters usually used to efficiently diagnose RA. The comparison of *κ*/*λ* hybrid antibodies with ACPA and RF showed that *κ*/*λ* hybrid antibody levels were positively correlated with RF (*r* = 0.53, *p* = 0.003) (Figure 3(a)) and that the *κ*/*λ* hybrid antibody levels were significantly higher in the ACPA-positive group (8.10 ± 4.12 AU/mL) than in the APCA-negative group (5.26 ± 2.06 AU/mL, *p* = 0.007) (Figure 3(b)). To further analyze the relationship between the *κ*/*λ* hybrid antibodies and inflammatory markers, RA-related inflammation factors, including IFN-*γ*, IL-10, TNF-*α*, D-dimer, and IL-6, were analyzed. The IFN-*γ* and D-dimer were significantly correlated with the *κ*/*λ* hybrid antibodies (*r* = 0.44, *p* = 0.02, Figure 3(c); *r* = 0.54, *p* < 0.0001, Figure 3(d), resp.). The IL-6 levels also displayed a trend toward positive correlation with the *κ*/*λ* hybrid antibodies, although the differences were not statistically meaningful. However, no correlations were observed with either IL-10 or TNF-*α*.

### 3.3. Serum *κ*/*λ* Hybrid Antibody Levels Correlate with the Parameters of Disease Activity

**Figure 4:**
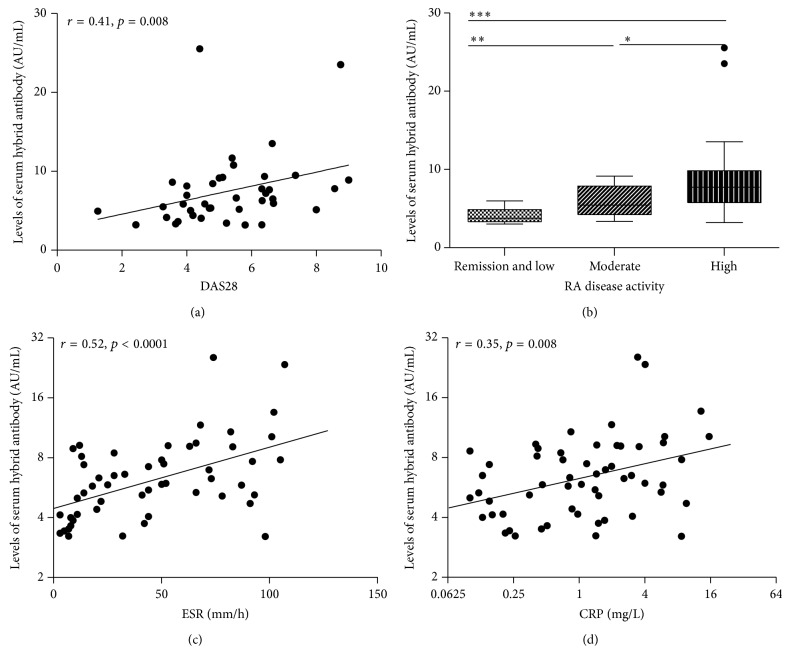
The relationship between serum levels of *κ*/*λ* hybrid antibody and disease activity in RA. (a) Levels of *κ*/*λ* hybrid antibody in the serum of RA patients positively correlated with DAS28 (*r* = 0.41, *p* = 0.008). (b) The distribution of *κ*/*λ* hybrid antibody in RA patients with different disease activity status; RA disease activity on the basis of DAS28 was defined as follows: remission and low = DAS28 < 3.2 (*n* = 10), moderate = DAS28 between 3.2 and 5.1 (*n* = 16), and high = DAS28 > 5.1 (*n* = 22); the *κ*/*λ* hybrid antibody levels were significantly higher in the high and moderate disease activity groups than in the low disease activity and remission groups (*p* = 0.008 and *p* = 0.0008, resp.); the differences of *κ*/*λ* hybrid antibody levels between groups were assessed using the Mann-Whitney *U* test, ^*∗*^ *p* < 0.05, ^*∗∗*^ *p* < 0.01, and ^*∗∗∗*^ *p* < 0.001. (c, d) Levels of *κ*/*λ* hybrid antibody in the serum of RA patients correlated with ESR (*r* = 0.52, *p* < 0.0001) and CRP (*r* = 0.35, *p* = 0.008). Concentrations of *κ*/*λ* hybrids antibody are presented in arbitrary units (AU/mL). Symbols represent individual subjects. Correlations were determined by Spearman's rank correlation coefficients with 95% confidence interval.

Subsequently, to examine whether serum *κ*/*λ* hybrid antibody levels are correlated with disease activity, DAS28, CRP, and ESR were investigated in the patients with ERA and established RA. By using the Spearman rank correlation test, *κ*/*λ* hybrid antibody levels were positively correlated with DAS28 in the RA patients (*r* = 0.41, *p* = 0.008; Figure 4(a)). The RA patients were then divided into four groups based on DAS28 as follows: clinical remission (*n* = 2), low disease activity (*n* = 8), moderate disease activity (*n* = 16), and high disease activity (*n* = 22). The remission group and low activity group were combined together due to the small sample size. The distribution of the *κ*/*λ* hybrid antibodies in the four groups is shown in Figure 4(b). The *κ*/*λ* hybrid antibody levels were significantly higher in the high and moderate disease activity groups than in the low disease activity and remission groups (*p* = 0.008 and *p* = 0.0008, resp.). We also found correlations between serum *κ*/*λ* hybrid antibody levels and ESR (*r* = 0.52, *p* < 0.0001; Figure 4(c)) and CRP level (*r* = 0.35, *p* = 0.008; Figure 4(d)).

### 3.4. Serum *κ*/*λ* Hybrid Antibody Levels Are Significantly Correlated with Serum IgG4 Level

Because *κ*/*λ* hybrid antibodies can form through the process of Fab-arm exchange, we were curious about whether *κ*/*λ* hybrid antibody levels are related to serum IgG4 (sIgG4) level. We measured sIgG4 levels in 58 RA patients; the mean sIgG4 concentration was 795 ± 710 mg/L, and 11 patients (three men and eight women) had elevated serum total IgG4 levels (>1,350 mg/L). Levels of *κ*/*λ* hybrid antibody in the elevated sIgG4 group (11.14 ± 7.14 AU/mL) were significantly higher than in the normal sIgG4 group (6.02 ± 2.12 AU/mL, *p* = 0.0047). Further analysis indicated that sIgG4 levels showed a positive correlation with the *κ*/*λ* hybrid antibody levels (*r* = 0.42, *p* = 0.001) (Figure 6).

## 4. Discussion

RA is a common systemic, inflammatory autoimmune disorder. Early treatment for patients with RA is crucial. The IgG4 *κ*/*λ* hybrid antibodies were studied in normal human serum, and they exhibited dynamic Fab-arm exchange [16]. In addition, a small fraction of *κ*/*λ* hybrid antibodies can be produced by dual receptor B cells. However, no study has investigated its clinical relevance to human diseases. In this study, we used a light-chain capture ELISA for *κ*/*λ* hybrid antibody detection in RA patients. Considering that *κ*/*λ* hybrid antibodies can be detected by using ELISA, analyzing their presence in RA serum would be intriguing because of minimally invasive and easy accessibility of samples for clinical use.

We report here, for the first time, the detection of *κ*/*λ* hybrid antibodies in RA and discuss their clinical values in autoimmune disease. The surprising finding is that increased serum *κ*/*λ* hybrid antibody levels were demonstrated in RA but not in HC. More importantly, *κ*/*λ* hybrid antibody levels were especially higher in the patients with ERA than in those with established RA and HC. These results indicate that elevated *κ*/*λ* hybrid antibody level is a biomarker of RA. OA is also a common cause of chronic joint disability, which affects the hand, hip, and knee joints of aging adults, and is difficult to distinguish from RA [23]. In our study, the *κ*/*λ* hybrid antibody levels were significantly higher in patients with ERA than in OA (*p* = 0.0003). This indicates that high levels of *κ*/*λ* hybrid antibodies are also a useful tool in distinguishing ERA from OA. Although the functional role and impact of *κ*/*λ* hybrid antibodies in living organisms remain unknown, we suggest that high serum *κ*/*λ* hybrid antibody levels in ERA patients may characterize an early stage of the disease.

The exact etiopathogenesis of RA is still uncertain. However, a range of inflammatory mediators produced by T cells, B cells, and macrophages are known to participate in the tissue-destructive process of the disease [24, 25]. Our study preliminarily discussed the relationship between levels of *κ*/*λ* hybrid antibody and the clinical inflammatory state of RA according to the results of inflammatory marker analysis, as determined based on ESR, CRP, IFN-*γ*, D-dimer, and IL-6 levels. These are well-studied inflammatory markers for a variety of acute and chronic inflammatory conditions and have been considered important in RA, as they reflect synovial inflammation [26–28]. For example, IFN-*γ* contributes to the pathogenesis of autoimmunity and IL-6 is associated with joint damage in RA [29]. D-dimer reflects the chronic inflammation in RA [30]. Increased ESR and CRP levels are markers of systemic inflammation and correlate with radiographic progression. In this study, serum CRP, ESR, IFN-*γ*, and D-dimer levels were significantly positively correlated with *κ*/*λ* hybrid antibodies in the RA patients. In addition, IL-6 level also showed a positive correlation trend. The positive association between *κ*/*λ* hybrid antibody, ESR, and CRP suggested that *κ*/*λ* hybrid antibody levels in RA possibly enable early discovery of ongoing inflammation. These results provide important evidence that *κ*/*λ* hybrid antibodies are not solely a result of disease development but rather act as a proinflammatory factor contributing to the tissue-destructive process of RA. Monitoring of *κ*/*λ* hybrid antibody levels may be a useful tool to determine the inflammatory status in RA. A theory that can explain these observations is still lacking. We speculate that hybrid antibodies could react with different antigen epitopes to form large immune complexes that may exacerbate inflammation.

In addition to the correlation analyses of these well-known inflammatory biomarkers, serum *κ*/*λ* hybrid antibody levels were compared with the autoantibody levels in RA. The two most important autoantibodies used for diagnosis are RF and ACPA. Serum RF is currently used for RA diagnosis because it is detected in 70–80% of RA patients, and high RF levels indicate poor prognosis [31]. ACPA is a recently identified highly specific and predictive biomarker of RA and is used as an indicator of joint destruction and a measurement for ERA [32, 33]. Our results showed an intimate relationship between high *κ*/*λ* hybrid antibodies, RF levels, and ACPA levels; being combined with the finding that levels of *κ*/*λ* hybrid antibody were also correlated with markers of RA disease activity suggested that *κ*/*λ* hybrid antibodies may act as a prognostic marker for RA.

**Figure 5:**
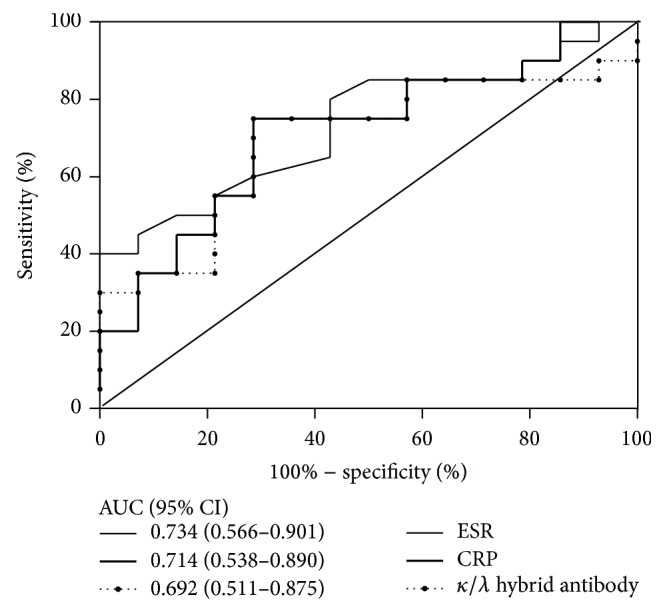
Receiver operating characteristic (ROC) curve analyses for *κ*/*λ* hybrid antibody, ESR, and CRP in discriminating moderate to high activity RA. The area under the curve (AUC) is presented. CI: confidence interval.

**Figure 6:**
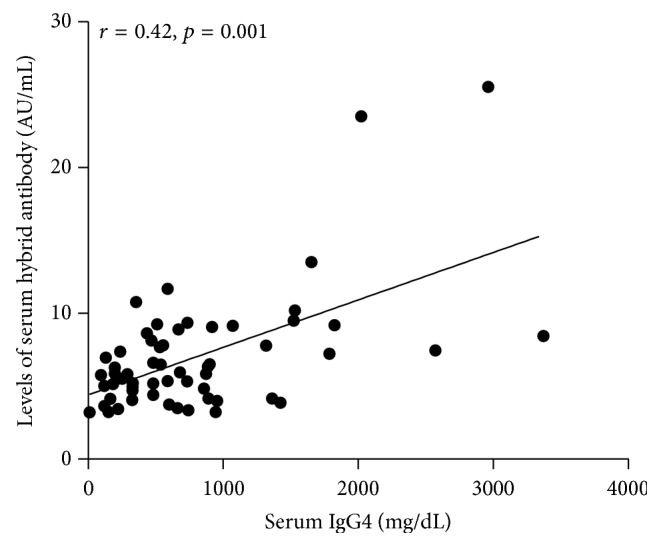
The relationship between serum levels of *κ*/*λ* hybrid antibody and serum IgG4 in patients with RA. Serum IgG4 (mg/dL) was measured in 58 RA patients using immunonephelometry. Levels of serum *κ*/*λ* hybrid antibody showed significant correlation with serum IgG4 (*r* = 0.43, *p* = 0.001). Concentrations of *κ*/*λ* hybrid antibody are presented in arbitrary units (AU/mL). Symbols represent individual subjects. Correlations were determined by Spearman's rank correlation coefficients with 95% confidence interval.

Next, we sought to determine whether *κ*/*λ* hybrid antibody levels were correlated with disease activity. In general, RA disease activity status can provide useful information for disease therapy. Surprisingly, the *κ*/*λ* hybrid antibody levels were shown to be positively associated with laboratory and clinical markers of RA disease activity, including CRP, ESR, and DAS28. The increased *κ*/*λ* hybrid antibody levels in the high and moderate activity groups indicate that higher *κ*/*λ* hybrid antibody levels represent a more severe disease process. We suggest *κ*/*λ* hybrid antibody levels as an auxiliary marker of disease activity in RA based on significant associations with CRP level, ESR, and DAS28. Since remission or low disease activity is the goal of modern therapeutic treat-to-target approaches [34], it would be meaningful to explore the differences between *κ*/*λ* hybrid antibody, CRP, and ESR in discriminating low disease activity from moderate and high activity in RA. However, due to the small number of RA patients in remission and low activity in our study (*n* = 10), as well as the presence of patients lacking laboratory data for ESR and/or CRP, it was hard to compare these parameters in low disease activity patients with others. Alternatively, we performed the ROC curve AUC analysis to determine the accuracy of *κ*/*λ* hybrid antibody, CRP, and ESR in discriminating moderate from high activity in RA. As shown in Figure 5, *κ*/*λ* hybrid antibody showed no superiority to ESR and CRP in discriminating the moderate from high activity RA. The AUC values for *κ*/*λ* hybrid antibody, CRP, and ESR were 0.692 (95% CI 0.511–0.875), 0.734 (95% CI 0.566–0.901), and 0.714 (95% CI 0.538–0.890), respectively. However, the use of hybrid antibody to discriminate low from moderate or high activity needs further investigation.

An interesting finding of this study is the moderate positive correlation between the *κ*/*λ* hybrid antibodies and serum total IgG4 levels. This phenomenon is consistent with previous reports that IgG4 is a dynamic molecule undergoing half-molecular exchange to produce IgG4 *κ*/*λ* hybrids [17, 35]. Actually, previous studies on the subclass distribution of ACPA and RF in RA indicate that IgG4 levels are conspicuously elevated, only secondarily to IgG1 [36–38]; consequently, serum positive for ACPA and RF may have a high incidence of half-molecule exchange. This is in agreement with our finding that *κ*/*λ* hybrid antibody levels are rich in ACPA-positive serum. In contrast, our data indicate that not just total sIgG4 levels but also other factors may contribute to the formation of *κ*/*λ* hybrid antibodies in RA. It is reported that glutathione (GSH) reduction in vitro can catalyze this process [39, 40]. Patients with RA demonstrated increased erythrocyte GSH-Px activities, unlike OA patients and healthy subjects, although opinions are controversial [41]. Furthermore, the dual receptor B cells can produce IgM hybrid antibodies that contain both *κ* and *λ* chains in the same molecule, which might not be limited to the IgG4 subclass. Taken together, the factors that favor the assembly of *κ*/*λ* hybrid antibody by Fab-arm exchange, such as oxidoreductive potential of the internal environment, and allelic inclusion of dual receptor B cells might jointly account for the production of *κ*/*λ* hybrid antibodies. Nevertheless, the exact role of these mechanisms needs to be carefully investigated in future research.

## 5. Conclusions

In summary, our results demonstrated for the first time that serum *κ*/*λ* hybrid antibody levels are markedly elevated in the patients with RA. Our data also support serum *κ*/*λ* hybrid antibody levels as an auxiliary marker of disease activity in RA. Moreover, we preliminarily discussed that elevated *κ*/*λ* hybrid antibody levels may reflect inflammatory conditions in RA. Next steps should include prospective studies on the possible role of *κ*/*λ* hybrid antibody in predicting and monitoring response early after initiation of treatment in large cohorts of RA patients.

## Figures and Tables

**Table 1:** Clinical characteristics of patients with early RA, established RA, and controls.

	Early RA	Established RA	OA	HC
Sex (F/M)	11/5	34/8	12/8	7/13
Age (years)	58.18 ± 16.19	56 ± 15.65	61.57 ± 13.11	43.21 ± 14.20
Disease duration	<6 months	8.81 ± 8.12 years	NA	0
Treatment				
Methotrexate	0	10	NA	0
Sulfasalazine	0	2	NA	0
Leflunomide	0	24	NA	0
Glucocorticoids	0	16	NA	0
Biologics	0	13	NA	0
Clinical character				
RF positivity (%)	62.5	82.9	NA	NA
ACPA positivity (%)	62.5	71.8	NA	NA
CRP (mg/L)	3.83 ± 4.57	2.15 ± 2.80	NA	NA
ESR (mm/h)	66.57 ± 26.61	39.02 ± 31.91	NA	NA
D-dimer (*μ*g/mL)	1426 ± 1598	604 ± 640	NA	NA

Data are expressed as the mean ± SD. ACPA, anti-citrullinated proteins antibodies; CRP, C reactive protein; ESR, erythrocyte sedimentation rate; HC, healthy controls; NA, not analyzed; OA, osteoarthritis; RA, rheumatoid arthritis; RF, rheumatoid factor.
